# Identification and Comparison of Peptides from Chickpea Protein Hydrolysates Using Either Bromelain or Gastrointestinal Enzymes and Their Relationship with Markers of Type 2 Diabetes and Bitterness

**DOI:** 10.3390/nu12123843

**Published:** 2020-12-16

**Authors:** Subhiksha Chandrasekaran, Diego Luna-Vital, Elvira Gonzalez de Mejia

**Affiliations:** Department of Food Science and Human Nutrition, University of Illinois at Urbana-Champaign, 228 ERML Bldg, 1201 W Gregory Drive, Urbana, IL 61801, USA; sc25@illinois.edu (S.C.); dieluna@illinois.edu (D.L.-V.)

**Keywords:** α-amylase, bitterness, bromelain, chickpea, DPP-IV, peptides, protein hydrolysates, type 2 diabetes mellitus

## Abstract

The chickpea (*Cicer arietinum* L.) is one of the most important pulses worldwide. The objective was to identify, compare and evaluate peptides from chickpea hydrolysates produced by two enzymatic treatments. The antidiabetic potential and bitterness of the peptides and induction of bitter receptors were identified in silico. Proteins were isolated from the Kabuli variety. Peptides were produced from the proteins using a simulated digestive system (pepsin/pancreatin, 1:50 Enzyme/Protein, E/P), and these peptides were compared with those produced via bromelain hydrolysis (1:50 E/P). The protein profiles, sequences and characteristics of the peptides were evaluated. The biochemical inhibition and molecular docking of dipeptidyl peptidase-IV (DPP-IV), α-amylase and α-glucosidase were also studied. The molecular docking identified peptides from enzymatic hydrolysis as inhibitors of DPP-IV. The high hydrophobicity of the peptides indicated the potential for bitterness. There was no correlation between peptide length and DPP-IV binding. Peptides sequenced from the pepsin/pancreatin hydrolysates, PHPATSGGGL and YVDGSGTPLT, had greater affinity for the DPP-IV catalytic site than the peptides from the bromelain hydrolysates. These results are in agreement with their biochemical inhibition, when considering the inhibition of sitagliptin (54.3 µg/mL) as a standard. The bitter receptors hTAS2R38, hTAS2R5, hTAS2R7 and hTAS2R14 were stimulated by most sequences, which could be beneficial in the treatment of type 2 diabetes. Chickpea hydrolysates could be utilized as functional ingredients to be included in the diet for the prevention of diabetes.

## 1. Introduction

The chickpea (*Cicer arietinum* L.), the third most produced legume worldwide, is grown on five continents [1]. A typical chickpea variety is expected to contain 59% carbohydrates, 29% protein, 5% oil, 3% fiber and 4% ash, which makes it an excellent source of plant-based protein. The chickpea has also been found to have beneficial effects in the prevention of diabetes, cardiovascular disease and cancer [2]. Additionally, chickpea protein isolated from chickpea flour and further digested by a variety of peptidases or acid–base methods has shown several bioactivities [3,4]. However, food products based on chickpea protein hydrolysates are not widely marketed. This is potentially due to the bitterness of the peptides produced upon digestion. Some protein hydrolysates have exhibited a bitter taste. Solutions to reduce and/or eliminate the bitterness of the peptides are currently needed, such that the bioactive properties of the chickpea protein hydrolysates can be appreciated [5].

The antidiabetic potential of chickpea protein hydrolysates needs further study. Diabetes mellitus is a chronic disease that results in an increase in blood glucose levels, usually as a result of the insufficient production of insulin by the pancreas. Approximately 400 million people worldwide live with type 2 diabetes mellitus (T2DM) [6]. Studies have shown that a plant-based diet can help with the management and prevention of T2DM [7]. T2DM is also often associated with cardiovascular diseases (CVDs) as comorbidities. The cost of diabetes management is steadily increasing and is estimated to reach up to USD 2.5 million in 2030 [8]. This indicates that there is an urgent need to find cost-efficient alternatives for managing and preventing T2DM.

Since there is a complex system in the control of glucose levels and insulin production in the body, there are multiple targets for foods and drugs to treat T2DM. Targets that have been explored in the past include dipeptidyl peptidase-IV (DPP-IV), α-amylase and α-glucosidase inhibitors [9,10,11]. The enzymes α-amylase and α-glucosidase participate in the digestion of starch from food; therefore, the inhibition of these markers decreases the glucose available for intestinal absorption and metabolism.

Regarding the sensory attributes of protein hydrolysate-based ingredients, it is also important to further study bitterness, since previous investigations on bitter compounds, from other foods, have proven beneficial effects in regulating diabetes. Food-derived compounds are expected to increase glucagon-like peptide-1 (GLP-1) secretion levels through a bitter-taste-receptor mechanism, which confers glucose sensitivity to *β*-cells and stimulates their proliferation [12]. Food-derived protein hydrolysates may also function as bitter-taste-receptor blockers, which could be beneficial in product development [13].

Previously published studies have largely focused on the enzymes present in the human digestive system, as well as alcalase and flavourzyme [4]. To the best of our knowledge, there are limited results for the sequences of peptides obtained from chickpeas produced using bromelain. Two patents, CN106957833A and CN107383159A, have listed bromelain as a potential enzyme used in the production of chickpea hydrolysates, but the sequences of the peptides are still unknown.

The objectives of this research were to identify and compare the antidiabetic potential of chickpea protein hydrolysates produced using two different enzymatic treatments, namely, a simulated gastrointestinal system with pepsin and pancreatin, and another system using bromelain. Protein profiles, peptide sequences and their in silico physicochemical characteristics were evaluated. The biochemical inhibition and molecular docking of DPP-IV, α-amylase and α-glucosidase were also studied. The potential role of the bitterness of the peptides present in the protein hydrolysates in the regulation of diabetes was evaluated using two databases.

## 2. Materials and Methods

### 2.1. Materials and Reagents

Kabuli chickpeas were provided by the Hebrew University of Jerusalem, Israel. Pepsin (MEROPS ID: A01.071), pancreatin and stem bromelain (EC 3.4.22.32) were purchased from Sigma-Aldrich (St. Louis, MO, USA). The chromogenic substrate Gly-Pro-pNA and dipeptidyl peptidase IV (EC 3.4.14.5), isolated from porcine kidney, were obtained from Sigma–Aldrich (St. Louis, MO, USA). α-amylase (EC 3.2.1.1), isolated from porcine pancreas, was also obtained from Sigma-Aldrich (St. Louis, MO, USA).

### 2.2. Preparation of Chickpea Protein Isolate

The procedure for isolating chickpea protein was adapted from a previous study [14]. The defatting step was omitted since fat did not dissolve in water at pH 11.5. Therefore, the discarded pellet, resulting at the end of the first round of centrifugation, contained the fat. The isoelectric point of the chickpea proteins was used to isolate them for further digestion.

### 2.3. Preparation of Chickpea Protein Hydrolysates

The chickpea protein isolate was dissolved in water at a 1:10 ratio. Hydrolysates were prepared with two enzymatic treatments. A simulated digestive system (pepsin/pancreatin, 1:50 enzyme/protein ratio, E/P) was compared to an exogenous enzyme, namely, bromelain (1:50 E/P). The hydrolysis procedure with pepsin, followed by hydrolysis with pancreatin to imitate the digestive system, was adapted from a previous study [15]. The hydrolysates were stored at 4 °C until further analysis. Previously established conditions for hydrolysis with bromelain were used [16]. The pH was measured at the beginning and at the end of the hydrolysis. Briefly, the pH of the solution containing the chickpea protein isolate was maintained at 5.5–6.5, which was the optimum pH range for hydrolysis, and the solution with added bromelain was placed in a water bath at 55 °C for 30 min. The enzyme was inactivated by boiling in water for 1 min. The solution was then stored at 4 °C until further analysis. Excess water from the solution was removed using a rotary evaporator at 45–60 °C and desalted using centrifugal ultrafiltration filters with a molecular cut-off below 3 kDa. The solutions were then freeze-dried for further analysis.

### 2.4. Peptide Sequencing

An established peptide-sequencing procedure was followed [17]. Briefly, peptides from both hydrolysates were analyzed using liquid chromatography–electrospray ionization-mass spectrometry/mass spectrometry. The mobile phase A was 95% water, 5% acetonitrile and 0.01% formic acid. Mobile phase B was 95% acetonitrile, 5% water and 0.01% formic acid. The volume of injection was 400 μL/min, and the wavelength of the photo diode array (PDA) detector was 280 nm. The MassLynx V4.1 (Milford, MA, USA) software was used to analyze the peptide sequences with >90% certainty for peptides with 8 or fewer amino acids, and with >60% certainty for peptides with >8 amino acids.

### 2.5. Molecular Docking

Molecular docking was performed using Autodock Vina (version 1.5.6, La Jolla, CA, USA). The structures of the predicted sequences were drawn using MarvinSketch (ChemAxon, Boston, MA, USA). The crystallographic structures of DPP-IV (PDB ID: 6B1E), α-amylase (PDB ID: 3BAJ) and α-glucosidase (PDB ID: 2QMJ) were retrieved from the Protein Data Base and were used to evaluate the antidiabetic potential of individual sequences found in the hydrolysates [18]. The docking position was determined using drugs previously docked onto the above-mentioned crystallographic systems. Using Discovery Studio V4.1 (Waltham, MA, USA), the ligands and water molecules were removed from the original template. Using Autodock Tools (version 1.5.6, La Jolla, CA, USA), the ligand was inputted into the template based on the active site of previously docked antidiabetic drugs [19]. The energy of the affinity with the active site of the enzyme was calculated using Autodock Vina [20]. Images of this interaction, which outline the amino acids in the peptide sequence and in the enzyme participating in the interaction, were generated using Discovery Studio V4.1.

### 2.6. Dipeptidyl Peptidase-IV Activity and α-Amylase Activity Assays

The DPP-IV activity assay was performed using the DPPIV-Glo kit (Promega, Madison, WI, USA) as per the manufacturer’s protocol. The chromogenic substrate Gly-Pro-pNA and DPP-IV (isolated from porcine kidney) were obtained from Sigma–Aldrich (St. Louis, MO, USA). Briefly, reconstituted luciferin detection reagent was mixed with DPPIV-Glo™ substrate in a 1:2000 ratio and incubated at room temperature for 30 min. Different concentrations of the hydrolysates were prepared by dissolving in a 100 mM Tris pH 8.0 reaction buffer. Then, 20 μL of sample, 25 μL of DPPIV-Glo™ and 5 μL of DPP-IV enzyme were added to a white 96-well plate for 30 min at 37 °C, and readings were taken with a luminescence detector. Diprotin A, a protease inhibitor (Sigma–Aldrich), was used as a positive control for inhibition [21].

The protocol for the α-amylase activity assay was adapted from a previous study [21]. Briefly, increasing concentrations of hydrolysate solutions were prepared based on the protein content of the hydrolysates. Acarbose (1 mM) was used as the positive control. All concentrations were added to 13 U/mL α-amylase solutions (type VI-B from porcine pancreas in 0.02 M sodium phosphate buffer, pH 6.9) and incubated in test tubes at 25 °C for 10 min. A 1% soluble starch solution in sodium phosphate buffer was then added and boiled for 15 min. After incubation for 10 min, the solutions were placed in a 100 °C water bath with 1 mL of dinitrosalicylic acid for 5 min. The mixtures were diluted with 10 mL of water, and the absorbance was read at 520 nm.

### 2.7. Properties of Peptides

Four databases were used to analyze the properties of the sequences obtained by hydrolysis. The presence of peptides in the chickpea proteins was confirmed using the BLAST^®^ tool (http://blast.ncbi.nlm.nih.gov/Blast.cgi, 1 September 2020). The physicochemical properties of the sequences produced by hydrolysis were evaluated using PepDraw (http://www.tulane.edu/~biochem/WW/PepDraw/index.html, 1 September 2020). BioPep was used to predict potential biological activity. It was also used to understand which segments of the obtained sequences were responsible for the bitter and umami tastes (http://www.uwm.edu.pl/biochemia/index.php/pl/biopep, 21 September 2020). The umami taste of the peptides was included since there is evidence that umami-tasting compounds can suppress bitterness [22]. BitterX [23] was used to understand which bitter receptors were likely to be activated by the obtained sequences. BitterX (Shanghai, China) uses previous data on bitter compounds and their stimulated taste receptors to predict if a new inputted compound will stimulate a specific bitter-taste receptor.

### 2.8. Statistical Analysis

All the assays and experimental procedures were performed in duplicate or triplicate to ensure reproducibility. The data are presented as mean ± standard deviation. The statistical analysis was performed using one-way ANOVA with Duncan’s multiple-range test. A *p*-value < 0.05 was considered as statistically significant.

## 3. Results

### 3.1. Peptide Sequencing of Chickpea Protein Hydrolysates

Multiple bioactivities were found by using the database BIOPEP for the sequenced peptides obtained in the hydrolysates produced with both the gastrointestinal (GI) enzymes and bromelain, respectively. Table 1 shows the amino acid sequences found in the hydrolysates produced using the simulated gastrointestinal digestive (GID) system; they typically contained at least one glycine molecule. The hydrophobicity of the peptides was between 3.6 and 20.3 kcal/mol. The isoelectric point ranged between pH 3.0 and 11.5. The lengths of the chains of amino acids ranged from 2 to 14 amino acids. The net charges of the peptides were between −1 and 2. Table 1 also presents the peptide sequences that may cause bitterness and umami tastes, based on the database BioPep, in the hydrolysates produced using the simulated GID, and their potential biological activity.

Table 2 shows that the sequences in the hydrolysates produced using bromelain also contained at least one molecule of glycine in the peptide sequence. The hydrophobicity of the peptides was between 10.2 and 28.3 kcal/mol. The isoelectric points ranged between pH 3.0 and 12.5. The lengths of the amino acid chains varied from 7 to 18 amino acids. The net charges of the sequences were between −2 and 3. Table 2 also presents the peptides potentially responsible for bitterness and umami tastes in the sequences of the hydrolysates produced by bromelain, and their potential biological activities.

Blank spaces indicate that there were no data on the umami taste of the amino acids, fragments of the sequence or entire sequence.

The parent proteins were investigated, and only two peptides, GKGSGAF and GKAAPGSGGGTKA, from the bromelain hydrolysate were identified to be derived from albumin and vicilin-like storage proteins, respectively. The other peptides were of metabolic origin.

### 3.2. Molecular Docking

Table 3 shows the energies of affinity with the active sites of DPP-IV, α-amylase and α-glucosidase for the sequences in the hydrolysates produced using the simulated GID. The sequences YVDGSGTPLT and PHPATSGGGL had the best affinity energies at −8.2 kcal/mol for the DPP-IV active site. The sequence SPQSPPFATPLW had the best energy of affinity at −8.4 kcal/mol for the active site of α-amylase. The sequence YVDGSGTPLT had the best affinity energy at −7.3 kcal/mol for the active site of α-glucosidase. Table 4 shows the energies of affinity with the active sites of DPP-IV, α-amylase and α-glucosidase for the sequences in the hydrolysates produced using bromelain. The sequence GKAAPGSGGGTKA had the most promising affinity energy at –7.3 kcal/mol for the active site of DPP-IV. The sequence KMTAGSGVT had the best energy of affinity for the active site of α-amylase at −7.1 kcal/mol, and the sequence GLTQGASLAGSGAPSPLF showed the most efficient affinity for α-glucosidase at −6.5 kcal/mol.

**Figure 1:**
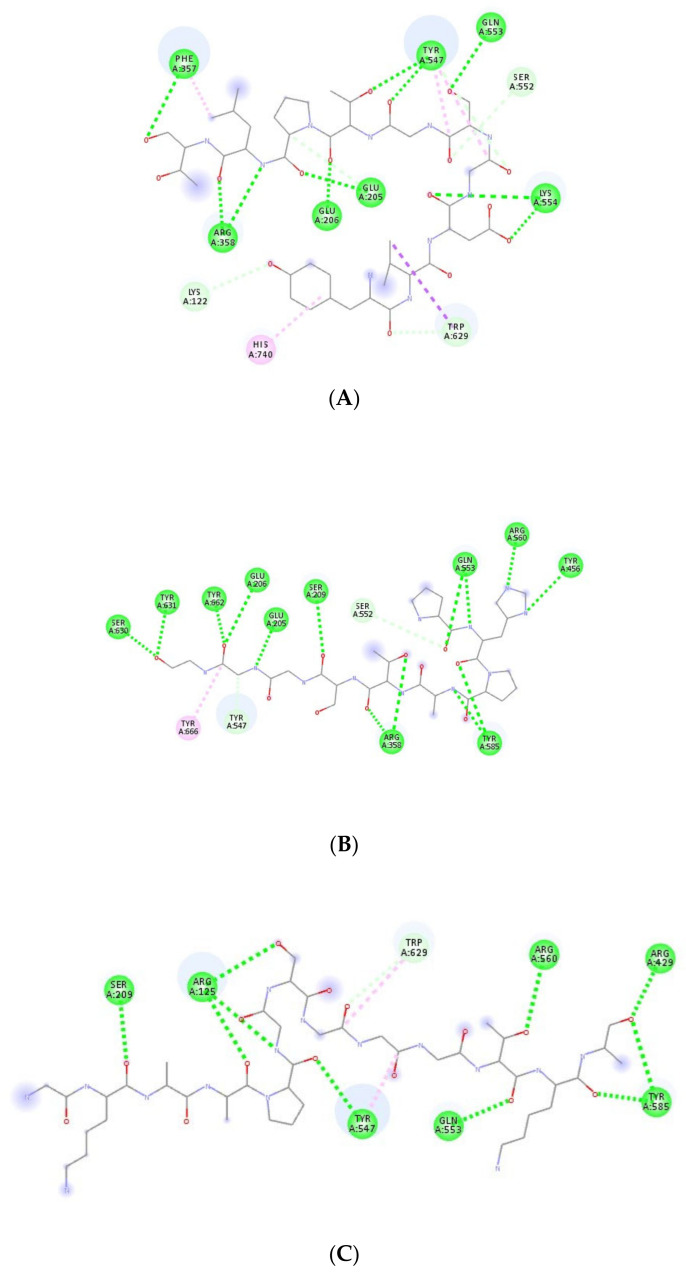
Best poses of the chickpea peptides (structure in gray) in the molecular-docking studies of sequences. (**A**) YVDGSGTPLT, (**B**) PHPATSGGGL and (**C**) GKAAPGSGGGTKA with dipeptidyl peptidase-IV (DPP-IV) obtained from Autodock Vina (version 1.5.6, La Jolla, CA, USA). Circles in green indicate amino acid residues from the catalytic site of DPP-IV.

Figure 1A–C show the interactions of the sequences YVDGSGTPLT, PHPATSGGGL and GKAAPGSGGGTKA with DPP-IV, respectively. Figure 1A shows that amino acids Phe357, Arg358, Lys122, His740, Glu206, Glu205, Lys554, Trp629, Ser552, Gln553 and Tyr547 participated in several interactions with the active site of DPP-IV. Figure 1B shows the amino acids Ser630, Tyr631, Tyr662, Glu206, Glu205, Ser209, Ser552, Tyr547, Tyr666, Arg358, Tyr585, Tyr456, Arg560 and Gln553 interacting with the active site of DPP-IV. Finally, Figure 1C shows the amino acids Ser209, Arg125, Tyr547, Trp629, Gln553, Arg560, Arg429 and Tyr585 interacting with the active site of DPP-IV. Tyr547 and Gln553 interacted with the active site for all three sequences that had the best energies of affinity with DPP-IV.

**Figure 2:**
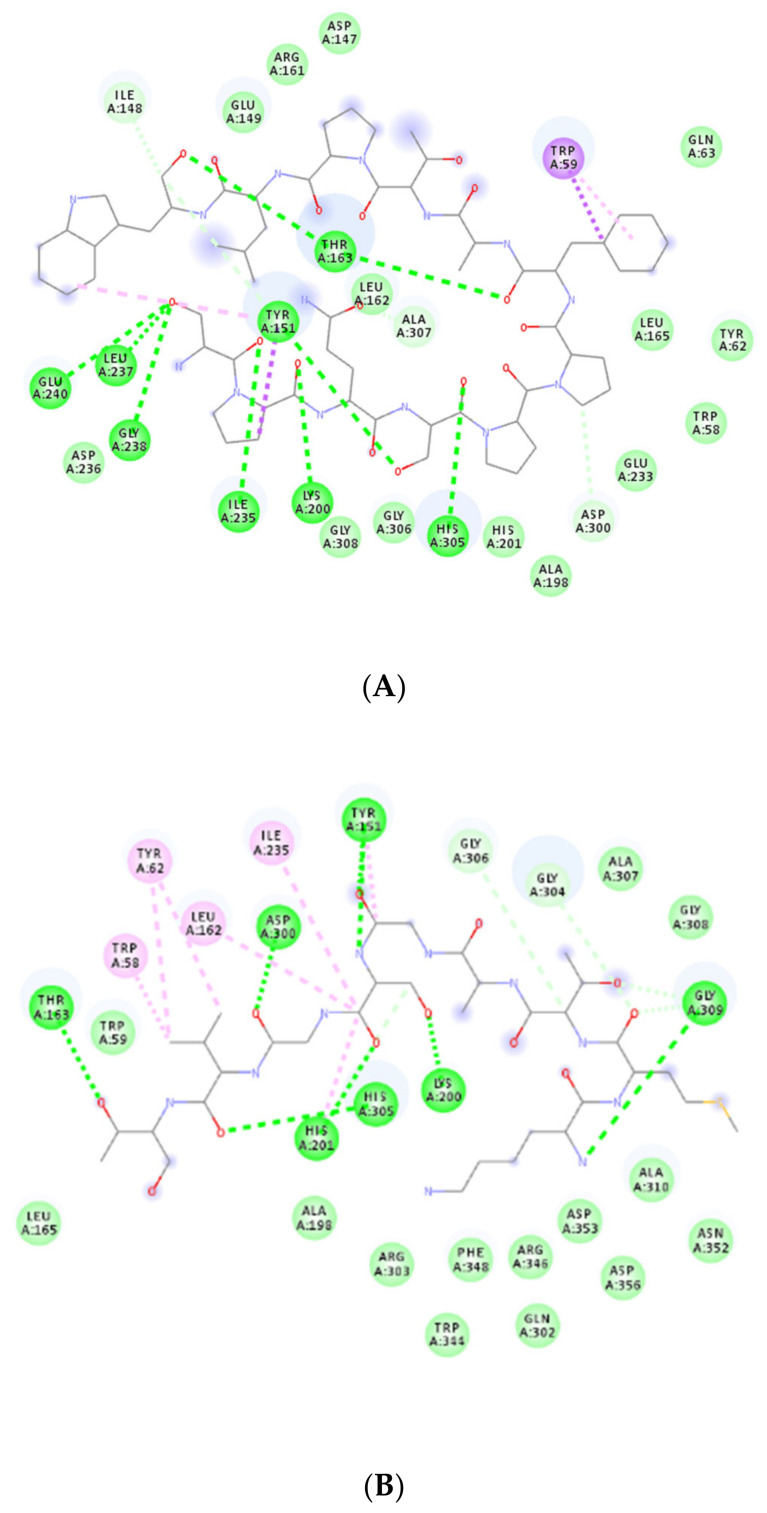
Best poses of the chickpea peptides (structure in gray) in the molecular-docking studies of sequences. (**A**) SPQSPPFATPLW and (**B**) KMTAGSGVT with α-amylase obtained from Autodock Vina (version 1.5.6, La Jolla, CA, USA). Circles in green indicate amino acid residues from the catalytic site of α-amylase.

Figure 2A,B show the interaction of the sequences SPQSPPFATPLW and KMTAGSGVT, respectively, with α-amylase. Figure 2A shows that the amino acids Ala307, Ile235, His305, Leu237, Gly238, Glu240, Tyr151, Lys200, Ile148, Thr163, Trp59, Asp236, Gly308, Gly306, His201, Ala198, Glu233, Trp58, Tyr62, Leu165, Gln63, Leu162, Glu149, Arg161, Asp147 and Asp300 interacted with the active site of α-amylase. Figure 2B depicts that amino acids Tyr151, Gly309, Lys200, Ile235, His201, Gly304, Thr163, Leu162, Asp300, Tyr62, Trp59, Gly306, Ala307, Gly308, Leu165, His305, Ala198, Arg303, Trp344, Phe348, Arg346, Gln302, Asp353, Asp356, Ala310, Asn352 and Trp58 interacted with the active site of α-amylase.

**Figure 3:**
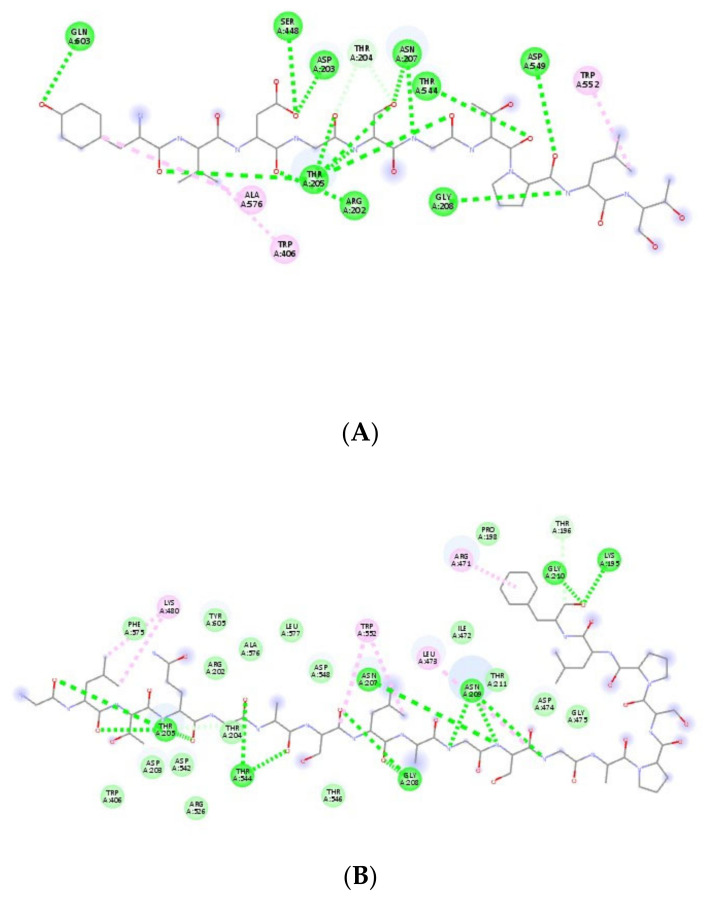
Best poses of the chickpea peptides (structure in gray) in the molecular-docking studies of sequences. (**A**) YVDGSGTPLT and (**B**) GLTQGASLAGSGAPSPLF with α-glucosidase obtained from Autodock Vina (version 1.5.6, La Jolla, CA, USA). Circles in green indicate amino acid residues from the catalytic site of α-amylase.

Figure 3A,B show the interactions of sequences YVDGSGTPLT and GLTQGASLAGSGAPSPLF with α-glucosidase, respectively. Figure 3A shows amino acids Gln603, Ala576, Trp406, Arg202, Thr205, Ser448, Asp203, Thr204, Asn207, Thr544, Asp549 Gly208 and Trp552 interacting with the active site of α-glucosidase. Figure 3B shows the amino acids Phe575, Lys480, Tyr605, Ala576, Leu577, Arg202, Asp548, Asn207, Ile472, Thr211, Pro198, Thr196, Thr544, Gly208, Arg471, Leu473, Thr205, Arg526, Trp552, Asn209, Asp474, Thr546, Gly475, Gly210, Lys195, Thr204, Trp406, Asp203 and Asp542 interacting with the active site of α-glucosidase. The structures of the peptides with the greatest energies of affinity for the three tested markers are presented in Figures S1 and S2.

### 3.3. DPP-IV Inhibitory Activities

Table S1 presents a summary of the activities of the most potent sequences found from the hydrolysis using a simulated GID and hydrolysis using bromelain. The peptides generated by the simulated GID system had greater affinity than the ones produced by bromelain for the DPP-IV catalytic site. These results are in agreement with the biochemical inhibition of DPP-IV—IC_50_ = 245 µg/mL (for the peptide fraction of the pepsin–pancreatin hydrolysate) and IC_50_ = 790 µg/mL (for the peptide fraction of the bromelain hydrolysate)—when compared with the drug sitagliptin (54.3 µg/mL). The percent inhibition of DPP-IV with different concentrations of the hydrolysates is shown in Figure S3.

α-amylase activity inhibition was 38.4 ± 1.4% relative to acarbose from the hydrolysate produced using the simulated GID system at a concentration of 10 mg/mL of protein. An 11.0 ± 0.8% inhibition relative to acarbose was observed from the hydrolysate produced using bromelain at the same protein concentration. The lengths of the peptides and the energies of interaction with DPP-IV, as well as the hydrophobicity of the peptides, were assessed for both enzymatic treatments. There was no correlation among the parameters in both enzymatic treatments.

Table S1 presents the most potent sequences isolated from the hydrolysis using simulated GID. PHPATSGGGL was one of the two most potent sequences in inhibiting DPP-IV, along with YVDGSGTPLT, which was also the most potent sequence for inhibiting α-glucosidase. SPQSPPFATPLW was the most potent sequence seen to inhibit α-amylase in molecular docking. Table S1 also presents the most potent sequences isolated from hydrolysis using bromelain. KMTAGSGVT was the most potent sequence inhibiting α-amylase. GKAAPGSGGGTKA was the most potent sequence inhibiting DPP-IV, and GLTQGASLAGSGAPSPLF the most potent sequence inhibiting α-glucosidase.

### 3.4. Bitterness-Related Properties of Sequences

BitterX (Shanghai, China) determined sequences that could be bitter. These sequences also had the ability to activate multiple bitter receptors. The bitter-taste receptors activated by the sequences generated using human digestive enzymes are listed in Table S2. The sequences generated using bromelain are listed in Table S3.

## 4. Discussion

Peptides from legume sources, such as chickpeas, could be used to generate functional ingredients with desirable functional properties and health benefits. We report from this research the amino acid sequences and potential sensory influences of chickpea peptides. The simulated GID system was compared alongside bromelain, an exogenous enzyme that can be used to produce ingredients for product development. A similar profile was obtained for chickpea protein hydrolysates using GID enzymes in silico [24]. Limited information is available on the profiles of peptides after protein hydrolysis using bromelain. The molecular weights of the peptides generated by bromelain ranged from 622.3 to 1670.8 g/mol.

Some peptide sequences from chickpea hydrolysates produced by GID enzymes have previously been identified under different growing conditions [25]. The exact sequences in this research did not match the sequences found in the hydrolysates produced using chickpeas grown in Israel. However, similar amino acids, namely, glycine, alanine, leucine, phenylalanine, tryptophan, lysine, glutamine, glutamic acid, serine, proline, valine, tyrosine, histidine, arginine, asparagine, aspartic acid and threonine, were part of the sequences. This could be due to the different origins of the materials.

Peptide sequences from chickpea hydrolysates produced using bromelain have previously been predicted [26]. The dipeptide fragments that were predicted matched 8 out of 12 sequences in the hydrolysates produced and identified in our investigation. However, sequences of three peptides or more did not match the sequences obtained from the in silico prediction study.

Certain amino acids, namely, Phe357, Arg358, His240, Glu206, Glu205, Trp629, Ser552, Tyr631, Tyr585, Ser209, Arg125, Tyr547 and Gln553, from chickpeas have not previously been found to interact with the active site in DPP-IV. The crystallographic structure of DPP-IV comprises a dimer with two domains forming a β-propeller domain with a cavity of approximately 30–45 Å between each monomer, where inhibitors bind in amino acid residues neighboring the catalytic site [27]. DPP-IV is considered an effective target for reducing blood sugar levels in patients with T2DM. These molecules promote enhanced insulin secretion in pancreatic β-cells in postprandial conditions.

The inhibitory activity on α-amylase and α-glucosidase of chickpea peptides was also evaluated. In agreement with our findings, the amino acids Asp300, His305, Trp59, Trp58, Tyr62, Ile235, Ala198, Tyr151, Glu233, Leu165, Lys200, Thr163, His201, Gly306, Asp356 and Glu240 were found to actively interact within the active site in α-amylase when investigating curcumin, acarbose, berberine and peptides from cumin seeds [28,29]. Interestingly, the interacting amino acids, when investigating peptides from pinto beans, did not match the ones obtained in this study, although pinto beans and chickpeas belong to the same family of plants [30].

No amino acid residues from previous studies matched the ones obtained in the molecular docking of the sequences from the chickpea hydrolysates on α-glucosidase. However, other variants of arginine, leucine, tyrosine and asparagine were found in previous studies of peptides [28].

The α-amylase inhibitory activity of chickpea peptides has not been reported before. A previous study on yellow pea protein showed that protein hydrolysates using individual human digestive enzymes produced a ranged between 27.7 and 30.5% in such activity. However, this was observed using a much lower concentration and with peptides of smaller molecular weights [31].

Inhibiting α-amylase and α-glucosidase is an effective strategy in the management of T2DM [32]. α-amylase is mainly present in the saliva and the pancreas, and it cleaves the alpha bonds of α-linked polysaccharides, for instance, in starch and glycogen. On the other hand, α-glucosidase cleaves the terminal non-reducing α-1,4 bonds to promote the release of single glucose molecules [33]. The hydrolysis of starch from foods into monomeric saccharides stimulates greater glucose absorption throughout the GI tract, and as a consequence, the levels of blood glucose increase.

It has been reported that in patients with obesity or T2DM, the inhibition of the above-mentioned enzymes by pharmacological inhibitors prevented dietary monomeric carbohydrates from being absorbed [34,35].

The bitterness of the sequences found in the present study can be hypothesized using the positions of certain amino acids and their hydrophobicity. The presence of six specific amino acids on the C-terminal, namely, arginine, proline, phenylalanine, leucine, isoleucine and tryptophan, has been established [36]. Twelve of the 20 predicted sequences in the GID hydrolysates contained the same amino acids on the C-terminal. Meanwhile, 7 of the 12 sequences in the hydrolysates produced using bromelain contained these amino acids on the C-terminal. Additionally, the presence of arginine, phenylalanine and leucine on the N-terminal may also indicate a potential bitter taste. Five of the 20 sequences in the hydrolysates produced when using human digestive enzymes contained these amino acids on the N-terminal, while 2 of the 12 amino acids in the hydrolysates produced using bromelain contained these amino acids on the N-terminal [36].

Bitter peptides from soybean protein hydrolysates have previously been reported [37]. Entire sequences from soybeans did not match the sequences found in chickpea protein hydrolysates, but a few fragments and similar amino acids were found. This is likely due to the fact that both foods belong to the Fabaceae family.

Previous publications have shown evidence that certain bitter receptors can be helpful in the management of T2DM. Specifically, hTAS2R5 and hTAS2R38 have been proven to result in the secretion of GLP-1 when activated by bitter compounds. The bitter receptors hTAS2R7 and hTAS2R14 have been reported to selectively increase the levels of cholecystokinin (CCK), a hormone that increases satiety. All of these receptors are likely to be activated by the sequences obtained in this study, thereby indicating a potential for further investigation into chickpea protein hydrolysates and T2DM [38].

There is scientific evidence that CCK plays a key role in the regulation of T2DM. CCK is primarily responsible for the regulation of energy intake. The cells that secrete CCK have been found to be co-localized with cells that produce GLP-1, a key regulator of blood glucose levels. Therefore, an increase in CCK secretion can be beneficial in the treatment of T2DM.

There is also evidence that adipogenesis can be affected through a bitter-taste-receptor mechanism. Specifically, mTAS2R106 has been shown to partly affect the pro-adipogenic effect induced by quinine, a known bitter compound [39]. Previous studies have established that adipocytes play a key role in the regulation of blood glucose levels, and there is an established correlation between T2DM and obesity [40]. Further investigation into the effect of chickpea protein hydrolysates on adipogenesis can also be performed in vitro and in vivo to determine the effects of the peptides beyond markers of T2DM found in pancreatic cells.

The information in this study could furthermore be used in product development to enhance the nutritional value of commercial foods. A recent study found that cross-linking chickpea proteins using transglutaminase results in emulsification properties [41]. Similar studies on the chickpea protein hydrolysates produced using GI enzymes and bromelain could be beneficial in producing a novel ingredient. Another study evaluated chickpea “milk” as a plant-based alternative to milk and hypothesized that the use of enzymes could be beneficial in modifying the final product and be a useful method in the optimization of the process [42].

The peptides from chickpeas found in the present investigation have the potential to inhibit relevant markers of T2DM. The information provided by this study could be used to develop new research aimed at evaluating the bitterness of these peptides in food matrices, as well as their antidiabetic potential in in vitro and in vivo studies.

## 5. Conclusions

For the first time, we have sequenced peptides that stimulate bitter receptors and show inhibition of enzymes related to type 2 diabetes, with implications for human health. Chickpea protein hydrolysates showed antidiabetic potential when specific peptide sequences were tested in molecular docking. Hydrolysis using bromelain yielded peptides that had greater molecular weights compared to hydrolysis using pepsin and pancreatin. The hydrolysates produced using GID showed better antidiabetic potential compared to the hydrolysates produced when using bromelain. Every predicted peptide sequence matched one of the many factors expected in bitter peptides. Therefore, it is still possible that they exhibit a bitter taste, and sensory studies are needed. An optimization of peptide generation using enzymatic treatments is needed, as is the further testing of pure peptide sequences for bitterness with multiple bitter receptors, specifically those established to have positive outcomes for helping T2DM. The results suggest that chickpea peptides could be incorporated as a value-added ingredient to design functional foods.

## Figures and Tables

**Table 1:** Peptide sequences found in chickpea protein hydrolysates produced using gastrointestinal (GID) enzymes.

Sequences	Molecular Mass (g/mol)	Isoelectric Point	Net Charge	Hydrophobicity (kcal/mol)	Length	Potential Bitterness Sequences	Potential Umami Taste Sequences	Potential Biological Activity
LR	287.2	10.7	1	8.5	2	L, R	-	Inhibits ACE, DPP-III, renin
PLLVE	563.3	3.0	−1	8.7	5	P, V, L, VE, LL, LV, PL	E, VE	Inhibits ACE, alpha-glucosidase, DPP-IV
SPKAGAGK	714.4	10.5	2	17.4	8	P, K, PK	-	Inhibits ACE, DPP-IV, DPP-III
HATGGGSGR	798.4	10.7	1	17.9	9	R, GR	-	Inhibits ACE, DPP-IV
PHPATSGGGL	892.4	7.8	0	13.9	10	P, L, GL, PA, GGL, GGGL	-	Inhibits ACE, DPP-IV, DPP-III
TPKASATAAL	929.5	10.1	1	12.6	10	P, K, L, PK	-	Inhibits ACE, DPP-IV, DPP-III
TLTTGTGGLL	932.5	5.4	0	8.6	10	L, LL, GL, GLL, GGL	-	Inhibits ACE, DPP-IV
YVDGSGTPLT	1008.5	3.1	−1	12.5	10	P, V, L, VD, PL, DG	D, VD, DG	Inhibits ACE, DPP-IV
TKTPGAGTSAGL	1059.5	10.1	1	15.3	12	P, K, L, GL, PG	-	Antiamnesic, antithrombotic, inhibits ACE, DPP-IV
KEGGGTGTGAAR	1060.5	9.8	1	23.4	12	R, K, EG, EGG	E, EG	Antioxidative, inhibits ACE, DPP-IV
STGPNAGGGAGGY	1064.5	5.4	0	16.8	13	P, GP, GY, GGY	-	Antiamnesic, antithrombotic, inhibits ACE DPP-IV
TLLFTELLF	1095.6	3.1	−1	3.6	9	F, L, LF, LL, EL, ELL	E, EL, TE	Antioxidative, inhibits ACE, DPP-IV, renin
KNGAAGPSTVAR	1127.6	11.5	2	17.6	12	R, P, K, V, GP, VA	-	Antiamnestic, antithrombotic, inhibits ACE, DPP-IV
LASEGASAATGAF	1151.5	3.2	−1	14.5	13	F, L, LA, EG, AF	E, EG	Inhibits ACE, DPP-IV
VLTSGAGSGAAALT	1174.6	5.5	0	11.8	14	V, L, VL	-	Antioxidative, inhibits ACE, DPP-IV
KNGLGAGAGAGSAR	1185.6	11.5	2	20.3	14	R, K, L, LG, GL, GLG	-	Inhibits ACE, DPP-IV
LSAHAGGTGATLW	1240.6	7.7	0	11.6	13	L, W, LW	-	Antioxidative, inhibits ACE, DPP-IV, renin
LDLARAGGCPTKN	1314.6	8.5	1	18.2	13	R, P, L, L, LD, DL, LA	D	Inhibits ACE, DPP-IV, DPP-III
SPQSPPFATPLW	1326.6	5.4	0	5.9	12	P, F, L, W, PP, PF, LW, PL, PPF	-	Antioxidative, inhibits ACE, alpha-glucosidase, DPP-IV, DPP-III, renin
LLSASMGSQLLSF	1352.7	5.5	0	4.8	13	F, L, LL	-	Inhibits ACE, DPP-IV, DPP-III, renin

Sequences were obtained using MassLynx V4.1 (Milford, MA, USA) software. Physicochemical properties of the sequences were obtained from PepDraw. Amino acid abbreviations: G—Glycine (Gly), P—Proline (Pro), A—Alanine (Ala), V—Valine (Val), L—Leucine (Leu), I—Isoleucine (Ile), M—Methionine (Met), C—Cysteine (Cys), F—Phenylalanine (Phe), Y—Tyrosine (Tyr), W—Tryptophan (Trp), H—Histidine (His), K—Lysine (Lys), R—Arginine (Arg), Q—Glutamine (Gln), N—Asparagine (Asn), E—Glutamic Acid (Glu), D—Aspartic Acid (Asp), S—Serine (Ser), T—Threonine (Thr). Potential causes of bitterness and umami taste were obtained from BioPep, as well as potential biological activity. Abbreviations of frequently listed bioactivities of peptides: ACE—Angiotensin converting enzyme, DPP—Dipeptidyl peptidase.

**Table 2:** Peptide sequences found in chickpea protein hydrolysates produced using bromelain.

Sequences	Molecular Mass (g/mol)	Isoelectric Point	Net Charge	Hydrophobicity (kcal/mol)	Length	Potential Causes of Bitterness	Potential Causes of Umami Taste	Potential Biological Activity
GKGSGAF	622.3	9.9	1	13.4	7	F, K, AF, KG	KG	Antioxidative, inhibit: ACE, DPP-IV
TRGTGGR	703.4	12.5	2	15.5	7	R, RG, GR	-	Inhibit: ACE, DPP-IV
KMTAGSGVT	850.4	9.8	1	13.3	9	V, K, GV	-	Inhibit: ACE, DPP-IV
KSGGGGGGTAVT	947.5	9.8	1	18.6	12	V, K	-	Inhibit: ACE DPP-IV
GKAAPGSGGGTKA	1057.6	10.7	2	21.6	13	P, K, PG	-	Antiamnestic, antithrombotic, inhibit: ACE, DPP-IV, DPP-III inhibitor
RASAAGGGGGGVSSR	1245.6	12.5	2	20.8	15	R, V, GV, GGV	-	HMG-CoA reductase, Inhibit: ACE, DPP-IV
GKGSSGTGAGGASVSGVT	1435.7	9.9	1	21.2	18	V, K, GV, KG	KG	Inhibit: ACE, DPP-IV
NKKSGAGGGSGAGKGGVA	1458.8	10.9	3	28.3	18	V, K, GV, KG, VA, GGV	KG	HMG-CoA reductase, inhibit: ACE, DPP-IV
LLGELCGSGNTVVEL	1502.8	3.0	−2	14.2	15	V, L, VE, GE, LL, LG, EL, VV, LLG	R, VV, VE, EL	Antioxidative, inhibit: ACE, alpha-glucosidase, DPP-IV, DPP-III
QNPLSSAAPTGAGKPY	1557.8	9.5	1	15.8	16	P, L, K, KP, PL	-	Antioxidative, inhibit: ACE, DPP-IV
GLTQGASLAGSGAPSPLF	1629.8	5.5	0	11.2	18	P, F, L, LF, GL, PL, LA, SLA	-	Inhibit: ACE, DPP-IV, DPP-III
AMMELGWSTSGEFLL	1670.8	3.0	−2	10.2	15	F, L, W, FL, GE, LL, LG, EL, EF, FLL	EL	Antioxidative, hypolipidemic, inhibit: ACE, DPP-IV, DPP-III

Sequences were found using MassLynx V4.1 (Milford, MA, USA). Physicochemical properties were obtained from PepDraw. Amino acid abbreviations: G—Glycine (Gly), P—Proline (Pro), A—Alanine (Ala), V—Valine (Val), L—Leucine (Leu), I—Isoleucine (Ile), M—Methionine (Met), C—Cysteine (Cys), F—Phenylalanine (Phe), Y—Tyrosine (Tyr), W—Tryptophan (Trp), H—Histidine (His), K—Lysine (Lys), R—Arginine (Arg), Q—Glutamine (Gln), N—Asparagine (Asn), E—Glutamic Acid (Glu), D—Aspartic Acid (Asp), S—Serine (Ser), T—Threonine (Thr). Potential causes of bitterness and umami tastes were obtained from BioPep, as well as potential biological activity. Abbreviations of frequently listed bioactivities of peptides: ACE—Angiotensin converting enzyme, DPP—Dipeptidyl peptidase.

**Table 3:** In silico-predicted interactions of peptides, sequenced after simulated gastrointestinal (GID) of chickpea protein, with dipeptidyl peptidase-IV (DPP-IV), α-amylase and α-glucosidase.

Sequences	Energy of Affinity with DPP-IV (kcal/mol)	Energy of Affinity with α-Amylase (kcal/mol)	Energy of Affinity with α-Glucosidase (kcal/mol)
LR	−5.0	−5.4	−4.5
PLLVE	−7.9	−7.6	−5.2
SPKAGAGK	−6.7	−7.1	−5.3
HATGGGSGR	−7.2	−6.7	−5.2
**PHPATSGGGL**	**−8.2**	−7.4	−7.1
TPKASATAAL	−7.7	−6.6	−6.8
TLTTGTGGLL	−7.8	−6.8	−5.6
**YVDGSGTPLT**	**−8.2**	−7.3	**−7.3**
TKTPGAGTSAGL	−7.1	−7.3	−5.8
KEGGGTGTGAAR	−7.2	−6.4	−5.6
STGPNAGGGAGGY	−7.6	−7.3	−6.3
TLLFTELLF	−7.3	−7.4	−6.0
KNGAAGPSTVAR	−6.9	−6.5	−5.7
LASEGASAATGAF	−7.3	−6.9	−6.1
VLTSGAGSGAAALT	−7.0	−6.7	−5.1
KNGLGAGAGAGSAR	−6.7	−6.2	−6.1
LSAHAGGTGATLW	−6.7	−7.9	−6.0
LDLARAGGCPTKN	−7.0	−6.3	−6.1
**SPQSPPFATPLW**	−7.0	**−8.4**	−7.2
LLSASMGSQLLSF	−6.6	−6.3	−5.9

Energy of affinity obtained using AutoDock Vina (version 1.5.6, La Jolla, CA, USA) and Discovery Studio V4.1 1 (Waltham, MA, USA). Enzyme structures were obtained from the Protein Data Bank [18]. Bold peptides indicate highest affinity. Amino acid abbreviations: G—Glycine (Gly), P—Proline (Pro), A—Alanine (Ala), V—Valine (Val), L—Leucine (Leu), I—Isoleucine (Ile), M—Methionine (Met), C—Cysteine (Cys), F—Phenylalanine (Phe), Y—Tyrosine (Tyr), W—Tryptophan (Trp), H—Histidine (His), K—Lysine (Lys), R—Arginine (Arg), Q—Glutamine (Gln), N—Asparagine (Asn), E—Glutamic Acid (Glu), D—Aspartic Acid (Asp), S—Serine (Ser), T—Threonine (Thr). The standard for DPP-IV docking was vildagliptin, which had an energy of affinity of −6.8 kcal/mol. The standard for both α-amylase and α-glucosidase was acarbose, which had energies of affinity of −9.6 and −7.1 kcal/mol, respectively. Bolded sequences and bolded numbers indicate the highest inhibition of the type 2 diabetes marker.

**Table 4:** In silico-predicted interactions of peptides, sequenced after digestion of chickpea protein using bromelain, with dipeptidyl peptidase-IV (DPP-IV), α-amylase and α-glucosidase.

Sequences	Energy of Affinity with DPP-IV (kcal/mol)	Energy of Affinity with α-Amylase	Energy of Affinity with α-Glucosidase
GKGSGAF	−6.8	−6.5	−5.5
TRGTGGR	−6.5	−6.7	−5.6
**KMTAGSGVT**	−6.8	**−7.1**	−6.2
KSGGGGGGTAVT	−6.9	−6.1	−5.5
**GKAAPGSGGGTKA**	**−7.3**	−6.5	−6.1
RASAAGGGGGGVSSR	−6.2	−6.5	−6.1
GKGSSGTGAGGASVSGVT	−6.5	−6.2	−5.6
NKKSGAGGGSGAGKGGVA	−5.8	−5.9	−6.1
LLGELCGSGNTVVEL	−6.8	−6.2	−5.2
QNPLSSAAPTGAGKPY	−6.8	−6.1	−6.4
**GLTQGASLAGSGAPSPLF**	−6.2	−6.4	**−6.5**
AMMELGWSTSGEFLL	−5.1	−5.9	−5.7

Energy of affinity obtained using Autodock Vina (version 1.5.6, La Jolla, CA, USA) and Discovery Studio V4.1 (Waltham, MA, USA). Enzyme structures were obtained from the Protein Data Bank [18]. Bold peptides indicate highest affinity. Amino acid abbreviations: G—Glycine (Gly), P—Proline (Pro), A—Alanine (Ala), V—Valine (Val), L—Leucine (Leu), I—Isoleucine (Ile), M—Methionine (Met), C—Cysteine (Cys), F—Phenylalanine (Phe), Y—Tyrosine (Tyr), W—Tryptophan (Trp), H—Histidine (His), K—Lysine (Lys), R—Arginine (Arg), Q—Glutamine (Gln), N—Asparagine (Asn), E—Glutamic Acid (Glu), D—Aspartic Acid (Asp), S—Serine (Ser), T—Threonine (Thr). The standard for DPP-IV docking was vildagliptin, which had an energy of affinity of −6.8 kcal/mol. The standard for both α-amylase and α-glucosidase was acarbose, which had energies of affinity of −9.6 and −7.1 kcal/mol, respectively. Bolded sequences and bolded numbers indicate the highest inhibition of the type 2 diabetes marker.

## Supplementary Materials

The following are available online at https://www.mdpi.com/2072-6643/12/12/3843/s1, Figure S1: Chemical structure of peptides (A) YVDGSGTPLT, (B) SPQSPPFATPLW and (C) PHPATSGGGL from chickpea protein hydrolysates produced using GID enzymes, obtained using PepDraw, Figure S2: Chemical structures of peptides (A) GKAAPGSGGGTKA, (B) KMTAGSGVT and (C) GLTQGASLAGSGAPSPLF from chickpea protein hydrolysates produced using bromelain, obtained using PepDraw, Figure S3: % DPP-IV inhibition by protein hydrolysates produced using gastrointestinal enzymes and bromelain, Table S1: A. Summary of the activities of the most potent sequences found in hydrolysis using a simulated GID and B. Hydrolysis using bromelain, Table S2: Bitter taste receptors that are activated by peptide sequences in chickpea protein hydrolysates generated using pepsin and pancreatin, Table S3: Bitter taste receptors that are activated by peptide sequences in chickpea protein hydrolysates generated using bromelain.
