# Supplementary material for: Identification and Comparison of Peptides from Chickpea Protein Hydrolysates Using Either Bromelain or Gastrointestinal Enzymes and Their Relationship with Markers of Type 2 Diabetes and Bitterness
Source: Nutrients. 2020 Dec 16;12(12):3843. doi: 10.3390/nu12123843 (PMC7765824; doi:10.3390/nu12123843)
Supplement: Supplementary file 1 [file nutrients-12-03843-s001.pdf]

Table S1.

A. Summary of the activities of the most potent sequences found in hydrolysis using a simulated GID

Peptide sequence Pepsin-Pancreatin (A)	Mass (g/mol)	Isoelectric point	Net charge	Hydrophobicity (kcal/mol)	Length (# of AA)	DPP-IV IC_{50} (μg/mol)	Inhibition α-amylase (%)
PHPATSGGGL	892.4	7.8	0	13.9	10	245	38.4 ± 1.4% relative to acarbose (at 10 mg/mL)
YVDGSGTPLT	1008.5	3.1	-1	12.5	10		
SPQSPPFATPLW	1326.6	5.4	0	5.9	12		

B. Hydrolysis using bromelain.

Peptide sequence Bromelain (B)	Mass (g/mol)	Isoelectric point	Net charge	Hydrophobicity (kcal/mol)	Length (# of AA)	DPP-IV IC_{50} (μg/Mol)	Inhibition α-amylase (%)
KMTAGSGVT	850.4	9.8	1	13.3	9	790	11.0 ± 0.8% relative to acarbose (at 10 mg/mL)
GKAAPGSGGGTKA	1057.6	10.7	2	21.6	13		
GLTQGASLAGSGAPSPLF	1557.8	9.5	1	15.8	16		

DPP-IV and α-amylase activity were measured with the hydrolysate mixture.

Table S2. Bitter taste receptors that are activated by peptide sequences in chickpea protein hydrolysates generated using pepsin and pancreatin.

Sequences	Activated Bitter Taste Receptors
LR	hTAS2R41, hTAS2R14, hTAS2R1, hTAS2R10
PLLVE	hTAS2R14, hTAS2R40, hTAS2R1, hTAS2R7, hTAS2R41, hTAS2R43, hTAS2R47, hTAS2R39, hTAS2R38, hTAS2R16, hTAS2R46, hTAS2R10, hTAS2R44, hTAS2R4
SPKAGAGK	hTAS2R1, hTAS2R14, hTAS2R16, hTAS2R41, hTAS2R40, hTAS2R7, hTAS2R47, hTAS2R39, hTAS2R38, hTAS2R43, hTAS2R10
HATGGGSGR	hTAS2R16, hTAS2R14, hTAS2R1, hTAS2R41, hTAS2R7, hTAS2R39, hTAS2R38, hTAS2R47, hTAS2R40
PHPATSGGGL	hTAS2R7, hTAS2R47, hTAS2R16, hTAS2R14, hTAS2R1, hTAS2R41, hTAS2R38, hTAS2R40, hTAS2R43, hTAS2R44, hTAS2R46, hTAS2R10, hTAS2R39
TPKASATAAL	hTAS2R40, hTAS2R7, hTAS2R14, hTAS2R41, hTAS2R1, hTAS2R47, hTAS2R16, hTAS2R39, hTAS2R43
TLTTGTGGLL	hTAS2R40, hTAS2R7, hTAS2R14, hTAS2R39, hTAS2R41, hTAS2R1, hTAS2R38, hTAS2R47, hTAS2R43, hTAS2R16
YVDGSGTPLT	hTAS2R7, hTAS2R40, hTAS2R14, hTAS2R47, hTAS2R41, hTAS2R38, hTAS2R1, hTAS2R39, hTAS2R43, hTAS2R16, hTAS2R44
TKTPGAGTSAGL	hTAS2R40, hTAS2R7, hTAS2R14, hTAS2R41, hTAS2R47, hTAS2R1, hTAS2R16, hTAS2R39, hTAS2R38, hTAS2R43
KEGGGTGTGAAR	hTAS2R7, hTAS2R40, hTAS2R14, hTAS2R41, hTAS2R1, hTAS2R1, hTAS2R39, hTAS2R16, hTAS2R47, hTAS2R38, hTAS2R43
STGPNAGGGAGGY	hTAS2R7, hTAS2R40, hTAS2R14, hTAS2R47, hTAS2R41, hTAS2R16, hTAS2R1, hTAS2R39, hTAS2R38, hTAS2R43, hTAS2R44
TLLFTELLF	hTAS2R40, hTAS2R5, hTAS2R41, hTAS2R39, hTAS2R7
KNGAAGPSTVAR	hTAS2R40, hTAS2R7, hTAS2R14, hTAS2R41, hTAS2R47, hTAS2R1, hTAS2R38, hTAS2R39, hTAS2R16, hTAS2R43
LASEGASAATGAF	hTAS2R40, hTAS2R7, hTAS2R41, hTAS2R39, hTAS2R14, hTAS2R5
VLTSGAGSGAAALT	hTAS2R40, hTAS2R7, hTAS2R41, hTAS2R14, hTAS2R39
KNGLGAGAGAGSAR	hTAS2R40, hTAS2R7, hTAS2R14, hTAS2R41, hTAS2R47, hTAS2R1, hTAS2R39, hTAS2R38, hTAS2R16, hTAS2R43
LSAHAGGTGATLW	hTAS2R40, hTAS2R7, hTAS2R41, hTAS2R47, hTAS2R14, hTAS2R5, hTAS2R39, hTAS2R38, hTAS2R43
LDLARAGGCPTKN	hTAS2R40, hTAS2R7, hTAS2R5, hTAS2R41, hTAS2R39, hTAS2R47
SPQSPPFATPLW	hTAS2R5, hTAS2R40
LLSASMGSQLLSF	hTAS2R40, hTAS2R5, hTAS2R41, hTAS2R7, hTAS2R39

Activated bitter receptors are listed in decreasing order of probability of activation. The minimum probability taken into account is 50%.

Table S3. Bitter taste receptors that are activated by peptide sequences in chickpea protein hydrolysates generated using bromelain.

Sequences	Activated Bitter taste receptors
GKGSGAF	hTAS2R16, hTS2R1, hTAS2R14, hTAS2R41, hTAS2R38, hTAS2R10, hTAS2R47, hTAS2R7, hTAS2R39, hTAS2R46, hTAS2R43, hTAS2R40, hTAS2R4, hTAS2R44
TRGTGGR	hTAS2R14, hTAS2R16, hTAS2R41, hTAS2R1, hTAS2R39, hTAS2R40, hTAS2R7, hTAS2R38
KMTAGSGVT	hTAS2R1, hTAS2R14, hTAS2R7, hTAS2R16, hTAS2R41, hTAS2R40, hTAS2R47, hTAS2R39, hTAS2R38, hTAS2R43, hTAS2R10
KSGGGGGGTAVT	hTAS2R14, hTAS2R7, hTAS2R40, hTAS2R1, hTAS2R16, hTAS2R41, hTAS2R39, hTAS2R47
GKAAPGSGGGTKA	hTAS2R7, hTAS2R40, hTAS2R14, hTAS2R47, hTAS2R41, hTAS2R16, hTAS2R1, hTAS2R38, hTAS2R39, hTAS2R43
RASAAGGGGGGVSSR	hTAS2R40, hTAS2R7, hTAS2R14, hTAS2R41, hTAS2R39, hTAS2R47, hTAS2R1, hTAS2R38, hTAS2R16

Activated bitter receptors are listed in decreasing order of probability of activation. The minimum probability taken into account is 50%. Some peptide sequences could not be evaluated using BitterX since the software can only process molecules that contain up to 250 atoms.

A)

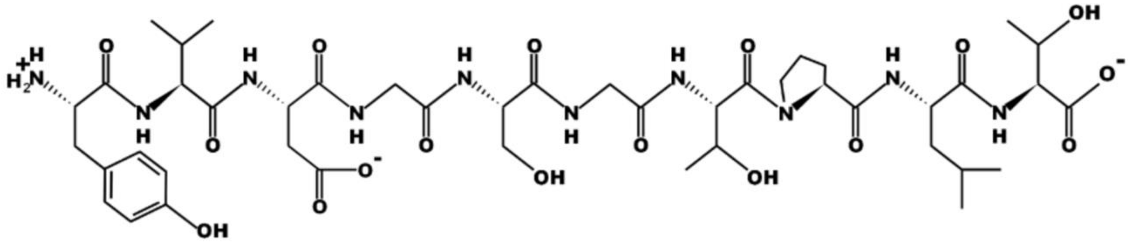

B)

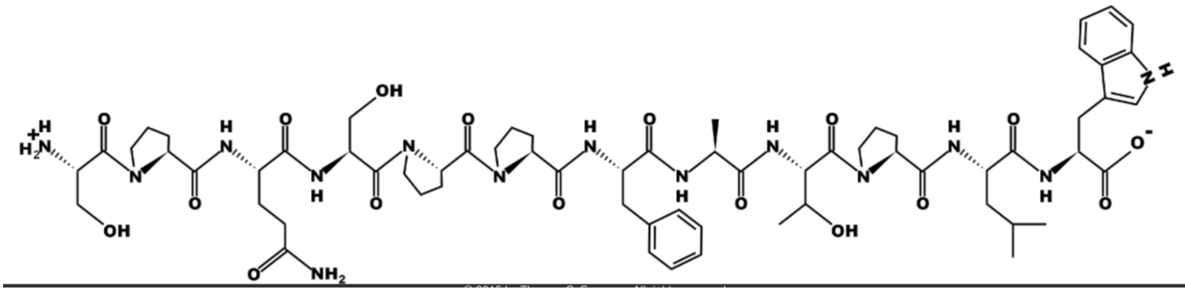

C)

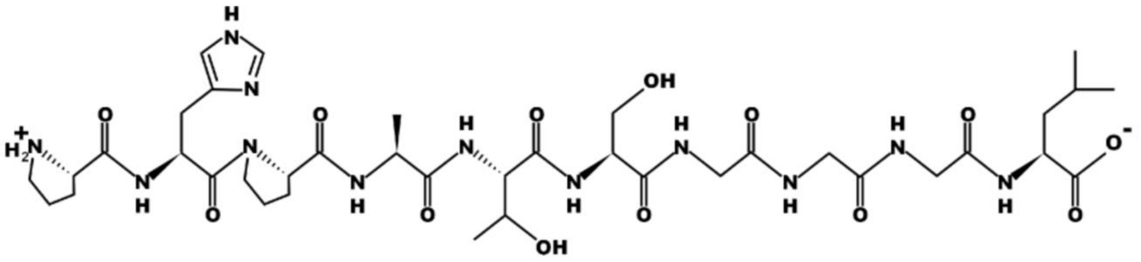

Figure S1. Chemical structure of peptides **A)** YVDGSGTPLT, **B)** SPQSPPFATPLW and **C)** PHPATSGGGL from chickpea protein hydrolysates produced using GID enzymes, obtained using PepDraw.

A)

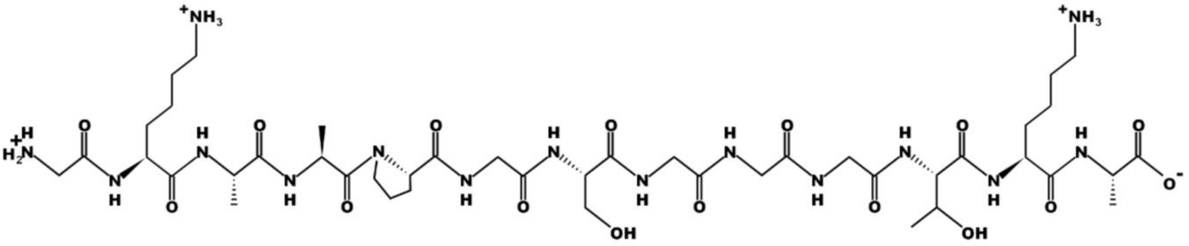

B)

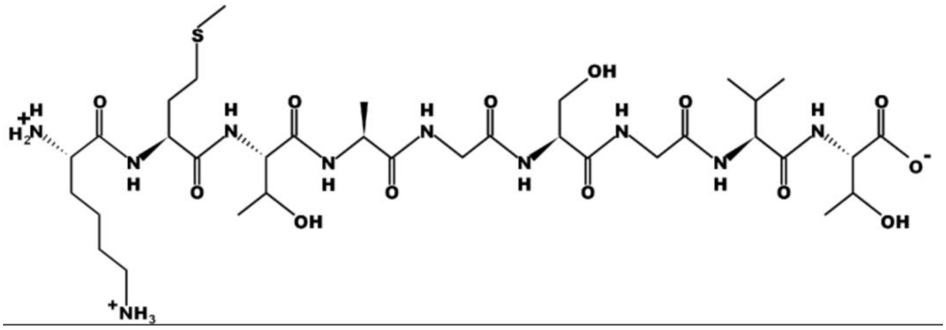

C)

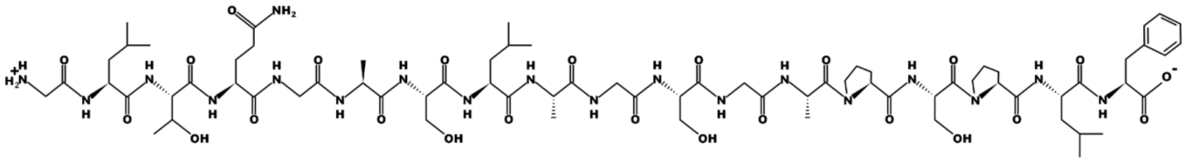

Figure S2. Chemical structure of peptides **A)** GKAAPGSGGGTKA **B)** KMTAGSGVT and **C)** GLTQGASLAGSGAPSPLF from chickpea protein hydrolysates produced using bromelain, obtained using PepDraw.

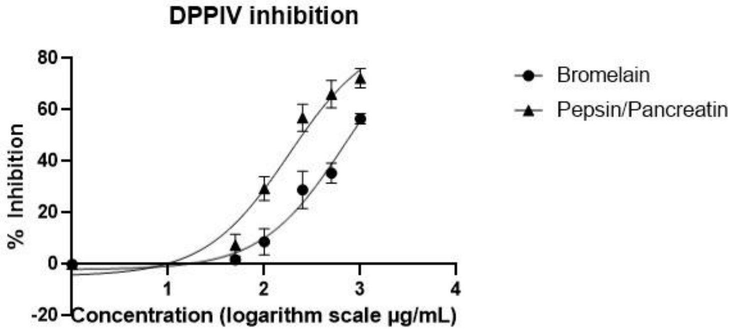

Figure S3. % DPP-IV inhibition by protein hydrolysates produced using gastrointestinal enzymes and bromelain.
